# The Relationship Between Public Risk Familiarity and Mental Health During the COVID-19 Epidemic: A Moderated Mediation Model

**DOI:** 10.3389/fpsyg.2022.945928

**Published:** 2022-07-05

**Authors:** Rui Qiu, Xia Zhu

**Affiliations:** Department of Military Medical Psychology, Air Force Medical University, Xi’an, China

**Keywords:** risk familiarity, mental health, mental toughness, SARS familiarity, psychological resilience

## Abstract

In order to explore, from the perspective of the social ecological model, the relationship and its mechanism linking public risk familiarity and mental health during the new coronary pneumonia epidemic, the new coronary pneumonia epidemic risk perception scale, psychological resilience scale, Chinese mental health scale, and SARS familiarity scale were used 741 members of the public were surveyed as research objects. The results show that: (1) When gender, age, and educational background are controlled, risk familiarity has a significant positive predictive effect on public mental health; (2) Risk familiarity predicts mental health through the mediating effect of mental toughness; (3) The mediating effect of mental toughness is moderated by the public’s familiarity with SARS. Specifically, for members of the public with low SARS familiarity, the indirect effect of risk familiarity on mental health through mental toughness is smaller than that for those with high SARS familiarity. The results of this study integrate psychological resilience into the theory of risk cognition, which has implications for the improvement of public mental health.

## Introduction

At the end of 2019, Corona Virus Disease 2019 (COVID-19), swept the globe, creating a public health crisis that poses serious threats and challenges to public safety, mental health, and social and economic development (Jiadong et al., 2020). According to Worldometer real-time statistics, as of late February 2022, there have been 42,218,754 confirmed cases of COVID-19 worldwide, and a total of 5,876,766 deaths. Studies have shown that COVID-19 has affected the public’s mental health, causing the development of behaviors such as maladaptation and reduced endurance, and also the development of negative emotions such as depression and anxiety (Fangfang et al., 2020). In the 27 provinces of China, the massive epidemic has caused varying degrees of psychological shock to the public (Zelong, 2020). With the promotion and dissemination of the new COVID-19 vaccines and the improvement of the government’s prevention and control measures, China’s epidemic preparedness has entered a normalized stage.

Prior studies on the impact of COVID-19 on the public’s mental health have mainly focused on the forms of the mental health problems (Shikang et al., 2020; Zhuoyan, 2020; Xia, 2022). However, few studies have examined the root causes of public psychological problems related to the outbreak of COVID-19, even though an understanding of the underlying causes of those problems can be an effective means to intervene in public mental health. Therefore, this study intends to investigate the relationship between COVID-19 risk perception and public mental health and its mechanism of action, in order to enrich the relevant research and provide a theoretical basis and support for scientific prevention and effective intervention for public psychological problems.

### Risk Perception and Mental Health

The idea of risk perception originated in the United States; it refers to an individual’s perception and understanding of various objective risks existing in the outside world through intuitive judgment and subjective feelings (Xiaofe and Liancang, 1995). Paul Slovic, an expert in risk decision-making, said: “Risk perception can provide a basis for understanding the public’s reaction to various risk events.” If we want to maintain and improve the safety and health of the public, we must explore and analyze people’s risk awareness and attitudes (Xiaofei and Rui, 2003). After the outbreak of the epidemic, the public’s perception of the COVID-19 epidemic as a risk event will be influenced by both the characteristics of the risk event (e.g., epidemic characteristics, transmission routes, effectiveness of diagnosis and treatment, and control types of COVID-19) and the public’s personal characteristics (e.g., gender, age, education level, temperament type, and environment) (Jinping et al., 2006).

Risk perception can affect mental health in many ways. First, different levels of risk perception will affect individuals’ emotional states (Xiaofei et al., 2005). Because of the wide and rapid spread of COVID-19, people often feel a serious threat from the disease, increasing their symptoms of stress, anxiety, and depression. Studies have shown that during the COVID-19 epidemic, the public’s fear of the disease, the proportion of sleep problems, and the detection rate of psychological distress have all increased. The public is troubled by the COVID-19 pandemic; the increasing time isolated at home and the limited range of activities have brought great challenges. If individuals cannot adapt to the changes brought about by these life events and change their perceptions accordingly, they will be prone to problems such as paranoia, emotional disorders, and somatization (Yuan and Min, 2020; Shanshan et al., 2021). Second, different levels of risk perception will affect individuals’ coping mentality (Yongtao and Qionglin, 2022). Studies have shown that the public’s coping attitude during the COVID-19 epidemic can be divided into three types. The first type is the positive coping attitude, in which individuals try to improve the surrounding environment to reduce the possibility and harm of risks. For example, researchers reduce the risk of COVID-19 infection by developing a COVID-19 vaccine. The second type is the self-protective mindset, in which individuals reduce the likelihood and harm of risks by enhancing their own resources and resilience. For example, individuals reduce the risk of COVID-19 through a healthy diet and regular exercise. The third type is the fearless risk-taking mentality, in which individuals ignore the dangerous situation they are in and engage in behaviors that threaten their own safety. When individuals are faced with sudden risk events, their original psychological balance will be broken.

If an individual perceives too much risk, they will fall into panic. Individual coping mentalities will also become polarized: one extreme is excessive paranoia, which may be manifested as compulsive hand washing and disinfection, while the other is a fearless mentality, which may be manifested as refusing to use protective equipment such as masks. These two mentalities are not conducive to the normal work and life of individuals, and constitute a state of psychological imbalance.

Sica et al. have shown that a high level of risk perception can trigger a series of adverse emotional experiences, which will in turn affect mental health (Gan et al., 2021; Hun, 2021; Sica et al., 2021; Li and Zhong, 2022). Conversely, Wu L. et al. (2021) have shown that a higher state of risk perception leads to a series of psychological response mechanisms, in which individuals establish new positive beliefs and cognitive styles and achieve growth in risk, thus improving their mental health (Dyer and Kolic, 2020; St John et al., 2020; Wu L. et al., 2021).

Based on the above analysis, this study will explore the relationship between risk perception and mental health in the context of the COVID-19 epidemic. Therefore, we propose the following hypothesis:

H1: *COVID-19 risk perception can positively predict mental health. The higher the level of risk perception, the higher the score on a mental health scale, and the worse the level of mental health*.

### Mediating Role of Resilience

Resilience is known to reduce anxiety levels; it is the ability to maintain or regain mental health despite adversity (Gemma et al., 2016). Resilience theory suggests that an individual with high resilience has the psychological strength needed to overcome difficulties, as well as the ability to improve and prevent negative psychological consequences such as anxiety (Skinner et al., 2020). Empirical studies have shown that, after controlling for gender and age, there is still a negative correlation between resilience and emotions such as tension and fear (Haddadi and Besharat, 2010; Bottomley et al., 2017; Singham et al., 2017; Kelly, 2021). Moreover, individuals with high resilience have stronger abilities such as emotional regulation, mastery motivation, and problem-solving ability. These enable them to maintain mental health and achieve prosocial development under adverse circumstances (Baum et al., 2021; Katie et al., 2021; Yau and Cecilia, 2021).

As a kind of perception of and cognition about an objective source of risk, risk perception will also affect the level of individual resilience. First, based on the factor-process integration model of resilience, an individual’s resilience level is affected by two factors: one is the influence of the induced event itself, and the other is the individual’s sensitivity to and awareness of the adversity event, as well as its severity. This second factor is thus risk perception, which will enhance the potential for the recovery of individual psychological function by re-evaluating and optimizing risk perception. An individual’s misperception of risk can reduce their acceptance of self and life, and their level of psychological resilience will thereby be reduced. Second, based on the protective factor model of resilience, protective factors play a positive role in producing resilience. Positive cognition of risk events constitutes a protective factor of positive cognitive style. An individual’s positive cognition of risk events can become a protective factor of psychological resilience, helping them to actively adapt to changes in the external environment and achieve good psychological development (Papageorgiou et al., 2018; Yankov et al., 2019; Biao et al., 2022).

The social-ecological model proposed by Guoliang et al. (2018) has been widely recognized. According to this theory, an individual’s level of mental health depends on the interactions between factors in different systems. Among them, an individual’s risk perception ability can be considered as a distal factor, while their level of resilience can be considered as a stable proximal factor. The distal factor of risk perception can affect mental health through the mediation of the proximal factor of resilience (Lin et al., 2017; Guoliang et al., 2018).

Based on the above analysis and the derivation of Hypothesis H1, we propose:

H2: *Risk perception positively predicts mental health through the mediation of resilience*.

### Regulation of Familiarity With Severe Acute Respiratory Syndrome

Resilience is the ability of individuals to grow and regenerate after encountering setbacks, and its impact on mental health is likely to be regulated by individual factors (Hailei and Wenxin, 2006). Familiarity with severe acute respiratory syndrome (SARS), an important individual cognitive difference, is likely to be a moderator between the two. SARS, which first appeared in Guangdong Province of China in 2002, spread globally in 2003. Like COVID-19, it became a public health event on a global scale. SARS has had a great impact on people’s work and life routines. Based on the theory of psychological antibodies, we know that when people have experienced traumatic events, they will gain corresponding experience and understanding, and when they face the corresponding events again, these prior experiences will play a guiding role, fluctuations in emotions will be reduced, and adaptation time will be shortened. Accordingly, we can infer that individuals who are more familiar with SARS have more experience and are more likely to make appropriate psychological and behavioral responses to the COVID-19 pandemic than those who are less familiar with SARS. The construction of this psychological immune response helps individuals improve their ability to resist pressure and develop tolerance, i.e., it helps them to improve their psychological resilience and improve their mental health level (Zhenqiu and Ying, 1992; Qingfen and Chongde, 2007; Changshan and Yuanyuan, 2012).

Therefore, we propose the following hypothesis:

H3: *Familiarity with Severe Acute Respiratory Syndrome (SARS) moderates risk perception and affects mental health through resilience, in that resilience is a better predictor of mental health for individuals with a higher familiarity with SARS than for individuals with a lower familiarity with SARS*.

In summary, based on the background of the COVID-19 pandemic, this study constructs a moderated mediation model to examine the relationship and its mechanism between public risk perception and mental health.

## Materials and Methods

### Object

In order to collect data to analyze the different types of public coping mentality, the research team used the convenience sampling method to select 741 members of the public as research subjects from December 27, 2021 to February 3, 2022.

After data cleaning and screening, 741 questionnaires were collected from 21 provinces and 92 cities in China, including 393 males (53.0%) and 348 (47.0%) females; 89 (12.0%) aged 20 and under, 204 (27.5%) aged 21–30, 155 (20.9%) aged 31–40, 159 (21.5 percent) aged 41–50, and 134 (18.1 percent) aged 51 and over. There were 123 people (16.6%) with a junior high school education or below, 192 people (25.9%) with a senior high school or junior college education, 266 people (35.9%) with a university education, and 160 people (21.6%) with a postgraduate education or above.

### Measuring Tools

#### COVID-19 Pandemic Risk Perception Scale

The Chinese version of the COVID-19 pandemic risk perception scale, as adapted by Ziluo and Zhiyu (2021) was used (Xiaonan and Jianxin, 2007). The scale includes 16 declarative questions about COVID-19, divided into four dimensions. Sample items are, “I understand the current development of COVID-19,” (familiarity dimension); “I am very afraid to go to the COVID-19 epidemic area,” (fear dimension); “The spread of the epidemic has exceeded human control,” (controllability dimension); and “The process of the spread of all viruses is also the process of their natural demise,” (naturalness dimension). A seven-point Likert scale was used to evaluate the subjects’ agreement with the questions, from 1 = *strongly disagree* to 7 = *strongly agree*. The total score was obtained by adding the scores of the individual reverse-scored items. The higher the total score, the higher the individual’s risk perception level. For this study, we selected the dimension of familiarity to measure the public’s risk perception of the COVID-19 pandemic. In this study, the Cronbach’s alpha coefficient of the familiarity dimension was 0.91, while the Cronbach’s alpha coefficient of the total scale was 0.71, and the construct validity was 0.87.

#### Resilience Scale

This study used the Chinese version of the Connor Davidson Resilience Scale (CD-RISC) as revised by Yu Xiaonan et al. The scale includes three dimensions of tenacity, strength, and optimism, with a total of 25 items, scored on a 5-point Likert scale. In Xiaonan and Jianxin (2007) tested the internal consistency of the Chinese version of the scale, and the result was 0.91 (Jisheng et al., 2006). In this study, the Cronbach’s alpha coefficient of the strength dimension was 0.94, the Cronbach’s alpha coefficient of the optimism dimension was 0.87, and the Cronbach’s alpha coefficient of the tenacity dimension was 0.96; the Cronbach’s alpha coefficient of the total scale was 0.98, and the construct validity was 0.99.

#### Chinese Mental Health Scale

This study used the Chinese Adult Mental Health Scale, developed by Jisheng et al. (2006), comprised of 10 subscales with 8 items each and 80 items in total. The scale has been proven to have good reliability and validity (Vajihe et al., 2022). Each item in the scale was scored by 5 grades, with 1 = *none*, 2 = *mild*, 3 = *moderate*, 4 = *severe*, and 5 = *severe* respectively. The higher the score, the more severe the individual’s mental health problems. Sample items are, “I feel that people are not friendly to me,” (interpersonal sensitivity subscale); “I feel that everything is difficult for me,” (psychological tolerance subscale); “I am afraid to deal with difficult things,” (adaptability subscale); “I feel people are unfair to me,” (psychological imbalance subscale); “My mood is high and low,” (emotional disorder sub-scale); “I’m upset about a lot of things,” (anxiety subscale); “I’m depressed,” (depression subscale); “I like to argue and argue with others,” (hostility subscale), “I don’t think most people can be trusted,” (paranoia subscale); and “My hands tremble when it comes to emergencies,” (somatization subscale). The Cronbach’s alphas of the individual subscales were: 0.97 for the interpersonal sensitivity subscale, 0.97 for the poor psychological endurance subscale, 0.96 for the poor adaptability subscale, 0.97 for the emotional dysregulation subscale, 0.97 for the anxiety subscale, 0.97 for the depression subscale, 0.97 for the hostility subscale, 0.97 for the paranoia subscale, and 0.96 for the somatization subscale. The Cronbach’s alpha coefficient of the total scale was 0.99, and the construct validity was 0.99.

## Results

### Correlation Analysis of COVID-19 Epidemic Risk Familiarity (COVID-19), Resilience, Severe Acute Respiratory Syndrome Familiarity, and Mental Health

Correlation analysis was conducted on the average scores for COVID-19 epidemic risk familiarity (COVID-19), resilience, SARS familiarity, and mental health.

The results show that there was a significant positive correlation between risk familiarity (COVID-19) and resilience, no significant correlation with SARS familiarity, and a significant negative correlation with mental health; there was no significant correlation between resilience and SARS familiarity, and a significant negative correlation between resilience and mental health; there was no significant correlation between SARS familiarity and mental health (see Table 1).

**TABLE 1:** Descriptive statistics and correlation coefficient matrix of the variables.

Variables	*M*	SD	1	2	3	4	5	6	7
1. Gender	1.47	0.50	1						
2. Age	3.06	1.30	0.06	1					
3. Academic qualifications	2.62	1.00	0.05	0.26**	1				
4. Risk familiarity (COVID-19)	4.97	1.49	0.07	0.25**	0.38**	1			
5. Resilience	3.74	0.95	0.01	0.36**	0.38**	0.79**	1		
6. Familiarity with SARS	2.03	0.98	–0.01	−0.18**	0.08*	–0.03	–0.02	1	
7. Mental health	2.51	1.27	−0.20**	–0.27	−0.35**	−0.09**	−0.16**	–0.03	1

M is mean, SD is standard deviation; N = 741; *p < 0.05, **p < 0.01.

### Test of the Mediating Effect of Mental Health on the Relationship Between COVID-19 Risk Familiarity (COVID-19) and Resilience

Mplus 8.7 was used to construct a structural equation model to explore the mediating role of resilience, and the items were packaged according to the dimensions of each variable to improve the fitting degree of the model. The bootstrap method was used to repeatedly sample 5,000 times to construct the 95% bias-corrected confidence interval of the mediating effect. If the confidence interval does not contain 0, the mediating effect is established.

The results of the model fitting indicators showed that χ^2^/*df* = 3.171, RMSEA = 0.054, CFI = 0.989, TLI = 0.987, SRMR = 0.024. Overall, the model fitting indicators met the requirements, indicating a good fit between the model and the data.

According to Table 2, which presents the regression analysis of risk familiarity (COVID-19), resilience, and mental health, risk familiarity (COVID-19) has a significant positive predictive effect on resilience, β = 0.847, *p* < 0.05; resilience significantly negatively predicts the score for mental health, β = −0.308, *p* < 0.05; and risk familiarity (COVID-19) positively predicts the score for mental health, β = 0.163, *p* < 0.05.

**TABLE 2:** Regression analysis of risk familiarity (COVID-19), resilience, and mental health.

Path	*b*	β	S.E.	*t*	*p*
Risk familiarity (COVID-19)→Resilience	0.564	0.847	0.019	30.278	0.001
Resilience→Mental health	–0.42	–0.308	0.097	–4.319	0.001
Risk familiarity (COVID-19)→Mental health	0.148	0.163	0.071	2.097	0.036

According to the analysis of the mediating effect of resilience between risk familiarity (COVID-19) and mental health (Table 3), the mediating effect of resilience on the relationship between risk familiarity (COVID-19) and mental health was −0.261, and the 95% confidence interval was [−0.387, −0.141].

**TABLE 3:** Analysis of the mediating effect of resilience between risk familiarity (COVID-19) and mental health.

Mediation path	Effect value	S.E.	*t*	*p*	LLCI	TLCI
Risk familiarity (COVID-19)→Resilience→Mental health	−0.261	0.063	−4.155	0.001	-0.387	−0.141

### Analysis of the Moderating Effect of Familiarity With Severe Acute Respiratory Syndrome

The PROCESS plug-in SPSS macro program was used to test the moderating effect of familiarity with SARS. The predictor variables in all equations were standardized and controlled for gender, age, and education. The mediation model test with regulation requires the construction of three different equations and the estimation of the parameters of the three regression equations. In the first equation, we need to estimate the overall effect of familiarity with SARS on mental health. In the second equation, we need to estimate the predictive effect of risk familiarity (COVID-19) on resilience. In the third equation, we need to estimate the moderating effect of familiarity with SARS on the relationship between resilience and mental health. The establishment of the mediating model effect with moderation requires three preconditions, as follows. Condition 1 requires that the total effect of risk familiarity (COVID-19) in Equation 1 on mental health be significant. Condition 2 requires that the predictive effect of risk familiarity (COVID-19) in Equation 2 on resilience be significant. Condition 3 requires that the main effect of resilience on mental health is significant in Equation 3 and that the interaction effect of familiarity with SARS and resilience must also be significant.

As shown in Table 4, the effect of Equation 1 is significant, indicating that risk familiarity (COVID-19) positively predicts mental health, and condition 1 is thus met. The effect of Equation 2 is significant, indicating that risk familiarity (COVID-19) positively predicts resilience, and condition 2 is thus met. Equation 3 is significant, showing that resilience negatively predicts the score for mental health, and the interaction term between familiarity with SARS and resilience is also significant.

**TABLE 4:** Test of the mediating effect of risk familiarity (COVID-19) on mental health.

Variables	Equation 1			Equation 2			Equation 3		
	(Validity criteria: Familiarity with SARS)			(Validity criteria: Mental toughness)			(Validity criteria: Mental health)		
	β	S.E.	*t*	β	S.E.	*t*	β	S.E.	*t*
Gender	−0.18	0.07	−5.53	−0.06	0.04	−2.59	−0.19	0.07	−5.75
Age	−0.20	0.03	−5.74	0.16	0.02	7.38	−0.18	0.03	−5.00
Academic qualifications	−0.32	0.04	−8.87	0.08	0.02	3.52	−0.30	0.04	−8.15
Risk familiarity (COVID-19)	0.10	0.04	2.63	0.73	0.02	31.52	0.16	0.06	2.93
Mental toughness							−0.10	0.06	−1.71
Familiarity with SARS							−0.03	0.03	−0.97
Familiarity with SARS × Mental toughness							−0.09	0.03	−2.72
*R* ^2^	0.19			0.67			0.20		
*F*	44.427***			379.423***			27.484***		

***p < 0.001.

**FIGURE 1:**
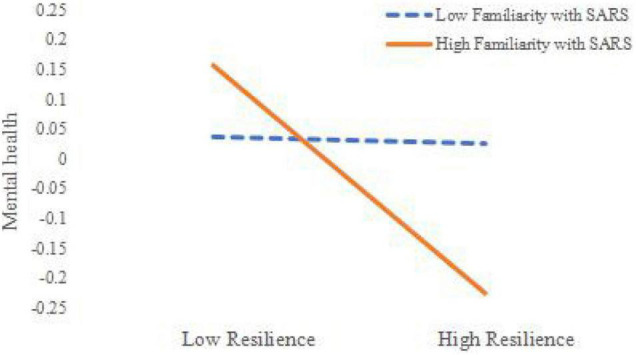
Moderating effect of familiarity with severe acute respiratory syndrome (SARS) on the relationship between resilience and mental health.

In order to explain the interactive effect of resilience and familiarity with SARS more clearly, we divided the participants into a low SARS familiarity group and a high SARS familiarity group according to the mean plus or minus one standard deviation. A simple slope test was performed and a simple effect analysis plot was drawn (see Figure 1).

The results in Figure 1 show that, for participants with a high degree of familiarity with SARS, resilience was a significant negative predictor of mental health scale scores; for participants with a low degree of familiarity with SARS, resilience was a weak negative predictor of mental health scale scores.

**FIGURE 2:**
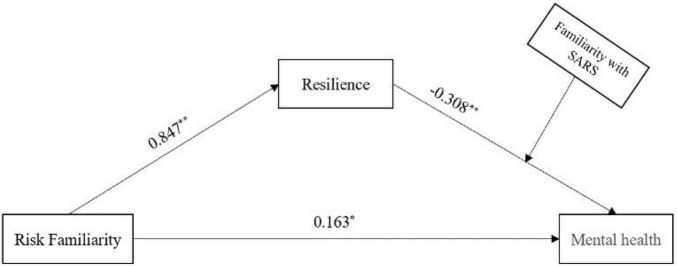
Holistic model of risk familiarity (COVID-19), resilience, familiarity with severe acute respiratory syndrome (SARS), and mental health. *p < 0.05, **p < 0.01.

Based on this, we can construct a holistic model of risk familiarity (COVID-19), resilience, familiarity with SARS, and mental health, as shown in Figure 2.

Based on the above analysis and the results shown in Figure 2, the process by which risk familiarity (COVID-19) influences mental health through resilience was modulated by the degree of familiarity with SARS. For individuals with low SARS familiarity, the indirect effect of risk familiarity (COVID-19) on mental health through resilience was relatively smaller than for those with high SARS familiarity.

## Discussion

From the perspective of risk perception and mental health, this study reveals the relationship between risk familiarity (COVID-19) and mental health and its mechanism. It was found that risk familiarity (COVID-19) affects the public’s mental health through the mediating effect of resilience. In addition, the latter part of this mediation model is moderated by familiarity with SARS. Resilience was found to be a better predictor of mental health than familiarity with SARS for those with a high degree of familiarity with SARS.

### Risk Familiarity (COVID-19) and Mental Health

This study found that risk familiarity (COVID-19) had a significant predictive effect on mental health. Prior studies have found that when individuals have a corresponding experience of risk or crisis events, they will form a graphical representation of the experience, thereby reducing the consumption of their cognitive resources. When they experience similar risks or crisis events again, they can produce more targeted solutions, which helps them to face risks. This in turn reduces their anxiety, depression, and other negative emotions and psychological stress (Jung et al., 2021; Wu J. L. et al., 2021; Hasegawa et al., 2022).

Mental health level is defined as the positive level of an individual’s psychological state based on the influences of their internal and external environments. The familiarity of risk has a positive impact on an individual’s mental health level. In particular, this study found that an individual’s familiarity with risk can significantly positively predict their mental health level, indicating that risk perception can predict and regulate mental health. Therefore, this study expands the perspective of public mental health research, by suggesting that when we study the influencing factors of mental health, we should not only pay attention to the degree of negative events encountered by individuals but also pay attention to their experience of risk events. As found in this study, a high familiarity with risk events may help the public to get a schema of coping with risk events. Given the difficulty of objectively changing risk events, subjective risk experience schema can help individuals to cope with negative events more actively and effectively.

### Mediating Role of Resilience

This study revealed that resilience plays a mediating role between risk familiarity (COVID-19) and mental health. These results support the social-ecological model of mental health, by showing that resilience is also an important mediating mechanism by which risk familiarity (COVID-19) affects mental health. Prior studies have shown that resilience has an important impact on individual mental health, and plays a central role in the regulation of mental health. If individuals have experienced risk events in real life, they will have a good idea of how to cope, which will enhance their mental resilience and help them maintain or recover their mental health in adversity (Labrague, 2021; Mojtahedi et al., 2021; Yaju, 2021; Yau and Cecilia, 2021). This experience of risk events will enhance the psychological strength individuals need to overcome difficulties and to prevent the negative psychological consequences caused by risk events, such as anxiety, paranoia, depression, and other emotional disorders. Therefore, the mediating role of resilience not only supports the social-ecological model of mental health but also integrates the theory of risk perception into the field of mental health, suggesting that risk familiarity (COVID-19) both also a proximal and remote factor of public mental health.

### Moderation by Familiarity With Severe Acute Respiratory Syndrome

This study found that familiarity with SARS moderates the mediating effect of resilience on the relationship between risk familiarity (COVID-19) and the latter half of public mental health. This is presumably because individuals who were more familiar with SARS had improved psychological resilience and had brought their experience of SARS to the COVID-19 pandemic. This makes it easier for them to make appropriate psychological and behavioral responses, and the construction of this psychological immune response ability can also help them to improve their psychological resilience, thereby improving their mental health (Cho and Kang, 2018; Wu et al., 2020; Yaju, 2021).

Although prior studies have clarified the relationship between resilience and mental health from both theoretical and empirical perspectives, few studies have examined the regulatory mechanism linking resilience and mental health. Prior studies have confirmed that the effect size of resilience on mental health is affected by individual differences. This study is the first to examine the regulatory mechanism of the effect of resilience on mental health from the perspective of a risk experience schema, which integrates individual experience, coping style, and personality characteristics, thereby expanding the model of mental health development and deepening the research on mental health problems. This conclusion also suggests that psychologists should pay attention to the differences in individuals’ experiences of risk events, and increase individuals’ experience and experience of risk events, which can effectively help them to improve their resilience and mental health. The adjustment of familiarity with SARS has opened up a new way of thinking about theoretical research and improvement of mental health, and the integration of resilience into the theory of risk perception is an important contribution of this paper.

### Research Limitations

This study also has the following limitations. First, this study is a cross-sectional study. The cross-sectional research methods and data used cannot accurately depict the changing trends of risk familiarity (COVID-19), resilience, and mental health over time, they cannot determine the direction of causality, and they can even lead to the distortion of the mediating effect ratio. Therefore, future studies must examine the causal relationship and long-term mechanism of risk familiarity (COVID-19), resilience, mental health, and familiarity with SARS over time using a longitudinal study. Second, this study only focuses on the relationship between resilience, risk familiarity (COVID-19), and mental health, but there may be other paths for this effect. Future research should continue to explore possible mediating variables and their internal mechanisms, to provide more reference for improving public mental health through multi-angle analysis of its influencing factors.

## Conclusion

In summary, this study found that:

(1) Risk familiarity (COVID-19) has a significant positive predictive effect on public mental health. Which supports the conclusion that risk familiarity (COVID-19), as a personal experience, has a positive effect on individual adaptation.
(2) Resilience plays a mediating role between risk familiarity (COVID-19) and mental health. Risk familiarity (COVID-19) enhances an individual’s resilience, and thereby improves their mental health.
(3) The indirect effect of risk familiarity (COVID-19) on mental health is mediated by the public’s familiarity with SARS through resilience. Specifically, the indirect effect was greater in individuals with high risk familiarity (COVID-19) than in those with low risk familiarity (COVID-19).

The results of this study have important theoretical significance and practical value for the scientific prevention of and active intervention into public mental health issues in the post-COVID era.

## Data Availability Statement

The original contributions presented in this study are included in the article/supplementary material, further inquiries can be directed to the corresponding author.

## Author Contributions

RQ wrote this manuscript. XZ did the supervision. Both authors contributed to the article and approved the submitted version.

## Conflict of Interest

The authors declare that the research was conducted in the absence of any commercial or financial relationships that could be construed as a potential conflict of interest.

## Publisher’s Note

All claims expressed in this article are solely those of the authors and do not necessarily represent those of their affiliated organizations, or those of the publisher, the editors and the reviewers. Any product that may be evaluated in this article, or claim that may be made by its manufacturer, is not guaranteed or endorsed by the publisher.

## References

[B1] Baum M. K. Tamargo J. A. Diaz-Martinez J. Delgado-Enciso I. Meade C. S. Kirk G. D. (2021). HIV, Psychological Resilience, and Substance Misuse During the COVID-19 Pandemic: A Multi-Cohort Study. *Drug and Alcohol Dependence* 231 109230. 10.1016/j.drugalcdep.2021.109230 34998257 PMC8704725

[B2] Biao F. Zonglong L. Kaixin W. Hongbo C. (2022). The relationship between life goals and mental toughness of college students: The multiple mediating effects of self-control and general self-efficacy. *Psychological Research* 15 78–85.

[B3] Bottomley J. S. Abrutyn S. Smigelsky M. A. Neimeyer R. A. (2017). Mental Health Symptomatology and Exposure to Non-Fatal Suicidal Behavior: Factors That Predict Vulnerability and Resilience Among College Students. *Archives of suicide research : official journal of the International Academy for Suicide Research* 22 596–614. 10.1080/13811118.2017.1387632 29111913

[B4] Changshan W. Yuanyuan H. (2012). On the role of experience in the construction of psychological immunity. *Journal of Tangshan Normal University* 34 149–151.

[B5] Cho H. H. Kang J. M. (2018). Effect of Resilience, Coping, and Mental Health on Burnout of Student Nurses. *Child Health Nursing Research* 24 199–207. 10.1016/j.nedt.2021.104852 33744814

[B6] Dyer J. Kolic B. (2020). Public risk perception and emotion on Twitter during the Covid-19 pandemic. *Applied Network Science* 5 99. 10.1007/s41109-020-00334-7 33344760 PMC7739810

[B7] Fangfang W. Shuhan M. Hanxue Y. Yue Q. Bin Z. (2020). “Psychological Typhoon Eye Effect” and “Ripple Effect”: Double perspective test of risk perception and anxiety characteristics of people in different COVID-19severityregions. *Acta Psychologica Sinica* 52 1087–1104. 10.3724/SP.J.1041.2020.01087

[B8] Gan Y. T. Zhang J. Quan Z. Y. (2021). Public perception of risk and coping response to COVID-19 in China: The moderating role of negative emotion. *Journal of Psychology in Africa* 31 117–123. 10.1080/14330237.2021.1903167

[B9] Gemma A. Merryn G. Karen H. (2016). What is resilience? An Integrative Review of the empirical literature. *Journal of advanced nursing* 72 980–1000. 10.1111/jan.12888 26748456

[B10] Guoliang Y. Jianliang L. Xie W. (2018). Ecosystem Theory and Adolescent Mental Health Education. *Educational Research* 39 110–117.

[B11] Haddadi P. Besharat M. A. (2010). Resilience, vulnerability and mental health. *Procedia - Social and Behavioral Sciences* 5 639–642. 10.1016/j.sbspro.2010.07.157

[B12] Hailei L. Wenxin Z. (2006). A Review of Mental Resilience Research. *Journal of Shandong Normal University (Humanities and Social Sciences Edition)* 149–152.

[B13] Hasegawa A. Oura I. Yamamoto T. Kunisato Y. Matsuda Y. Adachi M. (2022). Causes and consequences of stress generation: Longitudinal associations of negative events, aggressive behaviors, rumination, and depressive symptoms. *Current psychology (New Brunswick, N.J.)* 23 1–10. 10.1007/s12144-022-02859-9 35221638 PMC8864461

[B14] Hun C. D. (2021). The multifaceted impact of social media on risk, behavior, and negative emotions during the COVID-19 outbreak in South Korea. *Asian Journal of Communication* 31 10.1080/01292986.2021.1968447

[B15] Jiadong T. Bin S. Dianchun J. Bing Y. Jinping D. Cheng L. (2020). The global economy and challenges to China under the impact of the new crown pneumonia epidemic. *International Economic Review* 9–28.

[B16] Jinping L. Guangya Z. Hongqiang H. (2006). The structure, factors and research methods of risk perception. *Psychological Science* 370–372.

[B17] Jisheng W. Xiaoqing W. Xinhua D. (2006). The development and standardization of the Chinese Adult Mental Health Scale. *China Public Health* 137–138.

[B18] Jung S. J. Jeon Y. J. Choi K. W. Yang J. S. Chae J. H. Koenen K. C. (2021). Correlates of psychological resilience and risk: Prospective associations of self-reported and relative resilience with Connor-Davidson resilience scale, heart rate variability, and mental health indices. *Brain and Behavior* 11 e02091. 10.1002/brb3.2091 33638932 PMC8119814

[B19] Katie S. Mark C. Jacek G. (2021). Psychological resilience during COVID-19: a meta-review protocol. *BMJ open* 11 e051417. 10.1136/bmjopen-2021-051417 34145023 PMC8214992

[B20] Kelly C. (2021). Leveraging Longitudinal Pre-pandemic Data to Understand Mental Health Vulnerability and Resilience Among Young People During the Early Pandemic. *Biological psychiatry global open science* 1 244–245. 10.1016/j.bpsgos.2021.10.005 34927123 PMC8671761

[B21] Labrague L. J. (2021). Psychological resilience, coping behaviours, and social support among healthcare workers during the COVID-19 pandemic: A systematic review of quantitative studies. *Journal of nursing management* 29 1893–1905. 10.1111/jonm.13336 33843087 PMC8250179

[B22] Li P. P. Zhong F. (2022). Study on the Correlation Between Media Usage Frequency and Audiences’ Risk Perception, Emotion and Behavior. *Frontiers in Psychology* 12:822300. 10.3389/fpsyg.2021.822300 35126265 PMC8811358

[B23] Lin Y. Clough P. J. Welch J. Papageorgiou K. A. (2017). Individual differences in mental toughness associate with academic performance and income. *Personality and Individual Differences* 113 178–183. 10.1016/j.paid.2017.03.039

[B24] Mojtahedi D. Dagnall N. Denovan A. Clough P. Hull S. Canning D. (2021). The Relationship Between Mental Toughness, Job Loss, and Mental Health Issues During the COVID-19 Pandemic. *Frontiers in Psychiatry* 11:607246. 10.3389/fpsyt.2020.607246 33613333 PMC7886783

[B25] Papageorgiou K. A. Malanchini M. Denovan A. Clough P. J. Shakeshaft N. Schofield K. (2018). Longitudinal associations between narcissism, mental toughness and school achievement. *Personality and Individual Differences* 131 105–110. 10.1016/j.paid.2018.04.024

[B26] Qingfen H. Chongde L. (2007). The Influence of Experience Information and Covariation Information on Judging the Joint Action of Two Causes. *Applied Psychology* 11–18.

[B27] Shanshan W. Wenting Y. Qin H. (2021). Mental Health Status of Public and Medical Staff’s Relationship with Positive and Negative Emotion during Novel Coronavirus Disease Epidemic: An Online Survey. *Advances in Psychology* 11 10.12677/AP.2021.111019

[B28] Shikang Y. Chao W. Yong Y. Xie K. (2020). Analysis of Mental Health Status of Medical Students in the Late Stage of COVID-19 Epidemic. *Advances in Psychology* 10 1357–1362. 10.12677/AP.2020.109159

[B29] Sica C. Perkins E. R. Latzman R. D. Caudek C. Colpizzi I. Bottesi G. (2021). Psychopathy and COVID-19: Triarchic model traits as predictors of disease-risk perceptions and emotional well-being during a global pandemic. *Personality and Individual Differences* 176 110770. 10.1016/j.paid.2021.110770 33612905 PMC7879152

[B30] Singham T. Viding E. Schoeler T. Arseneault L. Ronald A. Cecil C. M. (2017). Concurrent and Longitudinal Contribution of Exposure to Bullying in Childhood to Mental Health: The Role of Vulnerability and Resilience. *JAMA Psychiatry* 74 1112–1119. 10.1001/jamapsychiatry.2017.2678 28979965 PMC5710218

[B31] Skinner E. A. Graham J. P. Brule H. Rickert N. Kindermann T. A. (2020). “I get knocked down but I get up again”: Integrative frameworks for studying the development of motivational resilience in school. *International Journal of Behavioral Development* 44 290–300. 10.1177/0165025420924122

[B32] St John F. A. V. Mason T. H. E. Bunnefeld N. (2020). The role of risk perception and affect in predicting support for conservation policy under rapid ecosystem change. *Conservation Science and Practice* 3 e316. 10.1111/csp2.316 33655201 PMC7116843

[B33] Vajihe A. Shahla A. Atefeh A. (2022). Health-care workers’ experience of stressors and adaptation strategies for COVID-19: A qualitative research. *Journal of Education and Health Promotion* 11 34. 35281372 10.4103/jehp.jehp_314_21 PMC8893097

[B34] Wu J. L. Hamilton J. L. Fresco D. M. Alloy L. B. Stange J. P. (2021). Decentering predicts attenuated Pperseverative tThought and Iinternalizing sSymptoms following stress exposure: A multi-level, multi-wave study. *Behaviour Research and Therapy* 152 104017. 10.1016/j.brat.2021.104017 35316616 PMC9007852

[B35] Wu L. Li X. Lyu H. (2021). The Relationship Between the Duration of Attention to Pandemic News and Depression During the Outbreak of Coronavirus Disease 2019: The Roles of Risk Perception and Future Time Perspective. *Frontiers in Psychology* 12:564284. 10.3389/fpsyg.2021.564284 33643120 PMC7905079

[B36] Wu Y. Yu W. Wu X. Wan H. Wang Y. Lu G. (2020). Psychological resilience and positive coping styles among Chinese undergraduate students: a cross-sectional study. *BMC psychology* 8:79. 10.1186/s40359-020-00444-y 32762769 PMC7406959

[B37] Xia Z. (2022). The Impact of Subjective Support on Mental Health under COVID-19: Analysis of Multiple Mediating Effects of Hope and Sense of Security. *Advances in Psychology* 12 10.12677/AP.2022.121025

[B38] Xiaofe X. Liancang X. (1995). Overview and theoretical framework of risk cognition research. *Psychological Dynamics* 17–22.

[B39] Xiaofei X. Rui Z. (2003). Risk Communication and Public Rationality. *Advances in Psychological Science* 11 375–381.

[B40] Xiaofei X. Zheng Rui X. Dongmei X. (2005). Analysis of Psychological Panic in SARS. *Journal of Peking University (Natural Science Edition)* 41 628–639.

[B41] Xiaonan Y. Jianxin Z. (2007). Comparison of Self-Resilience Scale and Connor-Davidson Resilience Scale. *Psychological Science* 1169–1171.

[B42] Yaju M. (2021). Effect of mental resilience of left-behind children on self-esteem and emotional processing bias and social coping styles. *Work (Reading, Mass.)* 69 559–571. 10.3233/WOR-213499 34120935

[B43] Yankov G. P. Davenport N. Sherman R. A. (2019). Locating mental toughness in factor models of personality. *Personality and Individual Differences* 151 109532. 10.22605/RRH5399 32237887

[B44] Yau T. Y. Y. Cecilia C. (2021). Internet Gaming Disorder, Risky Online Behaviour, and Mental Health in Hong Kong Adolescents: The Beneficial Role of Psychological Resilience. *Frontiers in Psychiatry* 12:722353. 10.3389/fpsyt.2021.722353 34721101 PMC8554051

[B45] Yongtao G. Qionglin F. (2022). Risk perception and coping response to COVID-19 mediated by positive and negative emotions: A study on Chinese college students. *PloS one* 17:e0262161. 10.1371/journal.pone.0262161 35061777 PMC8782533

[B46] Yuan L. Min D. (2020). Influence of Individual Public Crisis Ability on Anxiety under New Crown Epidemic Situation. *Advances in Psychology* 10 10.12677/AP.2020.1010169

[B47] Zelong Z. (2020). The remarkable advantages of the socialist system with Chinese characteristics from the perspective of fighting against the new crown pneumonia epidemic. *Theory Construction* 36 5–10.

[B48] Zhenqiu L. Ying Z. (1992). The role of recall experience in the process of memory: What does the experimental result of Ebbinghaus show? *Psychological Science* 23–26.

[B49] Zhuoyan W. (2020). Influence of Novel Coronavirus Pneumonia on Mental Status and Its Factors. *Advances in Psychology* 10 679–684. 10.12677/AP.2020.106083

[B50] Ziluo Y. Zhiyu X. (2021). The relationship between risk perception, psychological resilience and psychological stress response of the new crown epidemic. *Chinese Journal of Health Psychology* 1–10.

